# Bioengineering Approaches for Bladder Regeneration

**DOI:** 10.3390/ijms19061796

**Published:** 2018-06-17

**Authors:** Ángel Serrano-Aroca, César David Vera-Donoso, Victoria Moreno-Manzano

**Affiliations:** 1 Faculty of Veterinary and Experimental Sciences, Universidad Católica de Valencia San Vicente Mártir, 46001 Valencia, Spain; 2 Department of Urology, La Fe University and Polytechnic Hospital and Health Research Institute, Hospital La Fe, 46026 Valencia, Spain; cdveradonoso@gmail.com; 3 Neuronal Regeneration Lab, Centro de Investigación Príncipe Felipe, 46012 Valencia, Spain

**Keywords:** bladder regeneration, bioreactors, c, regenerative medicine, stem cells, scaffolds

## Abstract

Current clinical strategies for bladder reconstruction or substitution are associated to serious problems. Therefore, new alternative approaches are becoming more and more necessary. The purpose of this work is to review the state of the art of the current bioengineering advances and obstacles reported in bladder regeneration. Tissue bladder engineering requires an ideal engineered bladder scaffold composed of a biocompatible material suitable to sustain the mechanical forces necessary for bladder filling and emptying. In addition, an engineered bladder needs to reconstruct a compliant muscular wall and a highly specialized urothelium, well-orchestrated under control of autonomic and sensory innervations. Bioreactors play a very important role allowing cell growth and specialization into a tissue-engineered vascular construct within a physiological environment. Bioprinting technology is rapidly progressing, achieving the generation of custom-made structural supports using an increasing number of different polymers as ink with a high capacity of reproducibility. Although many promising results have been achieved, few of them have been tested with clinical success. This lack of satisfactory applications is a good reason to discourage researchers in this field and explains, somehow, the limited high-impact scientific production in this area during the last decade, emphasizing that still much more progress is required before bioengineered bladders become a commonplace in the clinical setting.

## 1. Introduction

Two decades have passed, waiting to see realized the realized promise of a regenerated bladder, while, for the last 80 years, the intestine has been used to functionally replace the bladder after its removal. What are the advances and what are the obstacles along this long and winding road? This review tries to answer this question.

**Figure 1:**
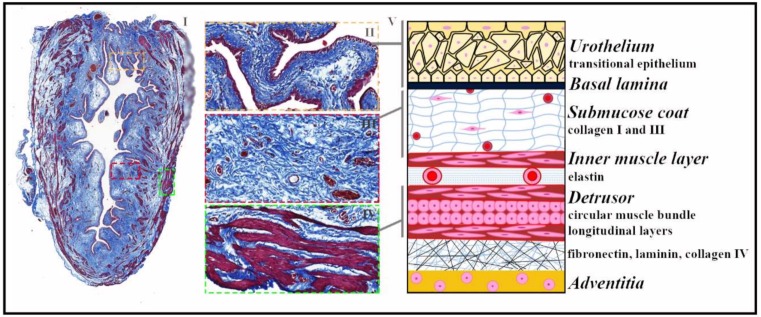
Urinary bladder histological organization in physiological conditions. (I–IV) Representative images of a longitudinal bladder section from an adult male rat after trichromic Masson staining. The adventitious layer (the outermost one), the urothelium (II), and the muscle bundles (IV) are stained in dark red, and the extracellular matrix of the submucosa is stained in blue (III). The location of the layers II, III, and IV is shown in image I by a corresponding colored frame with a dashed line; V: The illustration on the right shows the bladder organization in layers. Starting from the lumen, the bladder is composed of a transitional epithelium or urothelium formed by 4–5 layers of specialized cells supported by the basal lamina and followed by the submucosa coat, a loose connective tissue containing fibroblasts, blood vessels, and extracellular matrix and enriched in collagen I and III. Below, consecutive layers with perpendicular orientations of smooth muscle fibers form an inner muscle layer followed by the detrusor, characterized by smooth muscle fibers organized in circular and longitudinal layers. The adventitious layer of adipose tissue completes the structural organization.

Bladder dysfunction can be caused by several diseases or as a sequel of surgical interventions altering the normal cellular and extracellular matrix (ECM) compartments which accomplish the role of storage and voiding. The normal functioning urinary bladder is composed of two main parts: a compliant muscular wall and a highly specialized urothelium, well-orchestrated under the control of autonomic and sensory innervations. The specialized urothelium protects the upper urinary tract from urine reflux, pressure, and infection, providing a low-pressure and high-capacity reservoir (Figure 1). Repeated coordinated bladder contractions can be performed because of the specialized characteristics of the bladder’s smooth muscle fibers, blood vessels, and nerves. Furthermore, these contractions do not compromise the bladder storage capability nor the upper tract protection. Therefore, when this organ is damaged, all these properties must be taken into account in any attempt to replace the whole organ or part of it, with the goal of reproducing or recreating them individually, for a global functional recovery [1].

The urothelium, which is a transitional epithelium of endodermal origin, is necessary to prevent the passage of hypertonic urine to the blood and the exchange of toxic metabolites. About 90% of the urothelium is composed of basal cells, which are mitotically active cells, and 5% of the urothelium is populated by intermediate cells and superficial cells located in the suprabasal and luminal layers, respectively (Figure 1). In a state of homeostasis, adult urothelial cells are quiescent, with cell renewal rates of approximately 40 weeks; however, if the urothelium is damaged by an acute lesion, exposure to toxins, or a urinary pathology, a rapid process of exfoliation and regeneration is induced and completed in 72 h [1].

Numerous conditions, such as infection, cancer, trauma, inflammation, or iatrogenic injury, can cause the bladder to lose its ability to store and empty effectively. In addition, a progressive loss of bladder function resulting in a concomitant debilitating urinary incontinence or renal impairment can also be produced by congenital or neurologic disorders such as a spinal cord injury or spina bifida [2]. However, the most frequent cause of bladder removal is bladder cancer that requires a radical cystectomy. By incidence, this neoplasia is the sixth leading cancer in the European Union. Thus, 124,000 people are diagnosed, and more than 40,000 people die from the disease each year. Furthermore, the annual incidence is projected to increase to 219,000 by the year 2030, with two-fifths of this number due to the ageing of the European population [3].

**Figure 2:**
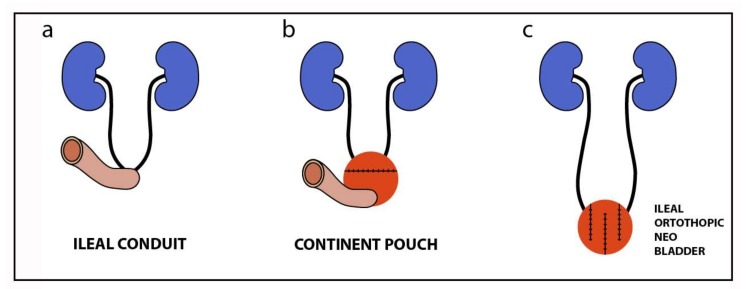
Types of urinary diversion currently performed after radical cystectomy: (**a**) abdominal diversion, such as an ureterocutaneostomy, colonic, or ileal conduit; (**b**) various forms of a continent pouch created using different segments of the gastrointestinal system and a cutaneous stoma; and (**c**) orthotopic urinary diversion with an intestinal segment with spherical configuration and anastomosis to the urethra (neobladder, orthotopic bladder substitution).

The standard radical cystectomy, i.e., the complete removal of the bladder, is nowadays the standard treatment for localized muscle-invasive bladder cancer [4]. In men, this operation also includes the removal of the prostate, seminal vesicles, distal ureters, and regional lymph nodes, and in women, the removal of the entire urethra and adjacent vagina, uterus, distal ureters, and regional lymph nodes [5]. However, controversy remains regarding age, radical cystectomy, and the type of urinary diversion [6] (see Figure 2). The selection of the primary therapy and type of urinary diversion is influenced by the performance status (PS) and age of a patient, and the cystectomy is usually reserved to younger patients without a concomitant disease and with a better PS. It has been reported that comorbidity is associated with adverse pathological and survival outcomes as a consequence of a radical cystectomy. When a conservative therapy, such as chemo- and radiotherapies, is not effective, for example after a bladder-sparing treatment and in the case of a non-urothelial carcinoma, salvage cystectomy is the most suitable solution. Sometimes, this clinical solution is also employed as a relieving intervention for fistula formation, pain, or recurrent macrohaematuria [7].

Nowadays, reconstructive urology is still facing the challenging problem of the replacement of the urinary bladder tissue with functional equivalents. The ileal conduit or neobladder generation are the gold standard approach for urinary diversion after performing a radical cystectomy [8,9] (Figure 2a,c). However, these techniques are associated with numerous complications, such as mucus production, electrolyte imbalances, and increased malignant transformation potential [10,11,12,13,14,15,16]. Early and late morbidity in up to 22% of the patients [17,18] and long-term complications, including diurnal (8–10%) and nocturnal (20–30%) incontinence, ureterointestinal stenosis (3–18%), metabolic disorders, and vitamin B12 deficiency, have been reported recently [19].

Nevertheless, bladder-preserving treatments such as radio- and chemotherapy are promoted, in the interest of the patients’ quality of life [20,21]. Conservative methods can be used to maximize patients’ safety and quality of life in the case of mild to moderate bladder dysfunctions and include behavioral training, clean intermittent catheterization, suprapubic catheterization, and pharmaceuticals treatments [22].

The most conservative treatments, such as bladder retraining or anticholinergic medications, typically do not produce a significant response in severe cases of bladder failure, seriously affecting the patient’s quality of life. In these kind of patients, an enterocystoplasty, which is a surgical bladder enlargement utilizing intestinal tissue, is currently performed [23]. The main aim of the surgical reconstruction is to increase bladder capacity and compliance, improving continence and reducing the intravesical storage pressure to protect the upper urinary tract. However, this clinical procedure fails to restore the emptying function and is associated with numerous complications [10,11,12,13,14,15,16,24], as already mentioned. Many alternative approaches [25,26,27] for bladder reconstruction have been attempted to find a practical and functional substitute material to the bowel segment and avoid the mentioned complications. However, only limited success has been achieved so far.

Although radical cystectomy can be performed through a robotic or open route, in most patients, urinary diversion is usually done by open surgery. The costs of this procedure differ from one country to another. Most of the costs and post-operative complications of the cystectomy are due to the derivation procedure that requires the implication of an intestinal segment. In the USA, the high costs of a neobladder operation and of an ileal conduit surgery after robotic cystectomy or open cystectomy, are compared in Table 1. In Canada, among 2759 patients, the average pre-surgery, radical cystectomy, and post-surgery costs were estimated at C$ 2284, C$18,345 and C$ 2095 (2014 Canadian dollars), respectively [28].

After cystectomy and urinary diversion, the hospital readmission rates are high, and the readmission costs are important. A very significant percentage (40%) of patients were readmitted to the hospital at least once within 90 days after robotic surgery, and 77% of the readmissions took place within 30 days. Twenty-seven patients, which represent a percentage of 11%, required two or more hospital readmissions. Readmissions occurred at a median of 13 days after the initial discharge. The direct costs produced by hospital-readmitted patients were 1.42 times of those for patients who did not require readmission. Readmissions for ileus problems contributed to the highest cost of readmission, although pelvic abscess, ureteral stricture, and sepsis presented the highest cost per hospitalization day [29].

In summary, currently, bladder replacement or repair with non-native urological tissue, such as autologous intestine, is performed as the clinical end-stage, when the primary treatment is not effective enough [31]. However, the utilization of the bowel tissue as a urological tissue substitute exhibits many problems [32], mainly due to the physiological functions of the intestinal epithelium, such as mucus secretion and digestion and absorption of nutrients, electrolytes, and water, which, thus, does not possess the suitable flexibility and impermeability to urine [33].

Therefore, new alternative strategies are needed to overcome all these problems and reduce the high economic impact of radical cystectomy and urinary diversion clinical solutions. Thus, much effort is currently focused on bioengineering approaches with polymers for urinary bladder regeneration, such as tissue engineering, bioreactors, and bioprinting.

## 2. Tissue Engineering for Urinary Bladder Regeneration

In the last two decades, tissue engineering has been focusing on bladder tissue reconstruction, and tremendous progress has been achieved in this field. A multidisciplinary bioengineering approach can be based primarily on the body’s natural regeneration ability and involve the use of a porous polymer matrix as a scaffold or of cell-seeded scaffolds, in which regeneration is even further promoted [34]. These advanced regenerative technologies have been studied in the quest to develop an efficient engineered bladder.

Tissue engineering for bladder substitution involves fostering cellular growth and maturation on a matrix, constituting a temporal or definitive scaffold, to accomplish the vesical functions. Bladder regeneration through tissue engineering offers important advantages, since it saves time in the operation room, allows the avoidance of gastrointestinal complications, and improves the patients’ quality of life. On the other hand, tissue engineering and regenerative medicine technologies for bladder replacement represent a very promising approach to develop novel therapeutics for other diverse lower urinary tract pathologies which do not necessarily requires complete bladder substitution [35,36]. Thus, many different animal models have been employed to evaluate the effectiveness of different cell-seeded scaffolds for bladder augmentation [37,38,39,40]. The idea of employing tissue engineering therapies for urinary bladder regeneration dates back to the 1950s. However, the necessary technologies were not ready yet for widespread clinical applications.

The scaffold is the key element supporting the vesical function. The new unspecialized bladder will be subjected to a dynamic mechanical and chemical stimulus during filling and emptying. The microenvironment generated by the matrix will influence the migration, invasion, proliferation, survival, or differentiation of the regenerating cells [37].

The polymeric biomaterials employed to engineer bladder tissue must fulfill several requirements, such as biocompatibility and adequate mechanical and chemical properties [41] in order to provide the structural support for several distinct cell layers and avoid a premature collapse of the hollow organ. The luminal side of the ideal biomaterial must provide a suitable surface for urothelial cell attachment serving as a barrier to avoid urine leakage into the surrounding tissue, and the visceral side must be capable to harbor the muscle cells, which must form unidirectional muscle tissue in defined layers and allow for rapid innervation and vascularization [42].

One of the most important goals is to engineer a bladder that can support engraftment avoiding a deleterious host immune response that can affect the functionality and durability of the allograft [43]. In addition, healing and vascularity [44], which are regulated by the inflammatory response, are also critical steps for a successful organ regeneration. Besides, it has been reported that macrophages can modify their phenotypic response to engineered foreign tissues to promote a proinflammatory or an anti-inflammatory response [45,46].

Engineered bladder constructs need to integrate into the body supporting urine storage. Furthermore, they need to possess contractile properties to allow physiologic voiding. Therefore, all these complex requirements still necessitate extensive research to obtain bladder constructs appropriate for clinical treatments.

Thus, many tissues and biomaterials of diverse nature, such as acellular tissues, natural polymers, synthetic polymers, and composites, have been used as bladder tissue substitutes and urinary bladder matrix scaffolds (see Table 2).

### 2.1. Natural Bladder Scaffolds

Natural acellular tissue scaffolds have shown to be a valuable option for whole tissue and organ regeneration, providing better functional outcomes than other tested synthetic biomaterials [62]. These type of polymeric scaffolds show a composition and a microstructure similar to the native tissue [63]. Furthermore, they retain chemical and biological cues, which are essential for tissue engineering strategies, because they constitute a more physiological environment for the target cells.

#### 2.1.1. Decellularization and Recellularization Technologies

Acellular tissue matrices can be prepared by mechanical or chemical decellularization processes, which consist of removing all the cellular components of human or animal tissues. Since these scaffolds for tissue and organ engineering present well-conserved and arranged structures of proteins and ECM components, the mechanical properties of these acellular matrices are very similar to those of the native tissue. These collagen-rich scaffolds slowly degrade after implantation while being replaced and remodeled by ECM proteins and supporting cell growth and regeneration, without evidence of immunogenic rejection [64].

Decellularization procedures have been applied to many types of tissues, such as dermis, liver, bladder, blood vessels, small intestinal submucosa, pericardium, trachea, ear, heart valves, and whole hearts [38,65,66,67,68,69,70], as well as musculoskeletal regions such as the temporomandibular joint [71]. Multiple ex vivo methods have been reported for the development of decellularized tissue scaffolds [72]. The main goal of the decellularization process is the complete removal of donor cells while preserving the remaining biologically active extracellular matrix scaffold, extracellular growth factors, and mechanical strength [73]. Common products employed for organ decellularization include acid–base agents, zwitterionic and non-ionic detergents, ionic or lysing detergents, hypotonic or hypertonic agents, enzymatic agents (usually trypsin and DNase), alcohols, and chelating agents. A broad array of agents used for sterilization, including peracetic acid, antibiotics, and radiation, and anticoagulants such as heparin sulfate are also necessary in this process. However, the ideal choice of agents and technique (perfusion, supercritical fluid exposure, pressure gradients, and mechanical agitation) used to apply these agents depends on the kind of organ to be decellularized and its specific properties: tissue cellularity, density, lipid composition, and thickness [74,75,76]. An optimal decellularization protocol must be composed of a combination of physical, chemical, and enzymatic techniques [77]. For whole porcine bladder decellularization, Yang et al. defined an efficient method which simultaneously preserved the extracellular bioactive factors and further improved the recellularization process [78]. The intact porcine bladders were decellularized by using distention techniques. Bladder distension can reduce the thickness of the bladder wall and thus facilitate cell removal [79,80,81], being the urinary bladder a highly compliant and distensible vessel for holding and releasing urine [82].

The first step for an efficient decellularization involved urothelium removal by an enzymatic treatment with 0.25% trypsin/0.038% Ethylenediamine tetra-acetic acid (EDTA), applied through a catheter fixed into the bladder neck and with continued physical agitation for 2 h at room temperature and followed by three washes with ice-cold phosphate-buffered saline (PBS). During the subsequent procedures, the bladder was kept distended with a washing buffer and completely immersed in the same solution. Then, the bladder was incubated in an ice-cold hypotonic Tris buffer and, after three washes with ice-cold PBS, it was incubated for 24 h at room temperature in hypertonic Tris buffer containing 1.0% Triton X-100. The final goal of organ decellularization is the removal of all foreign cells and immunogenic compounds, while optimizing and preserving the biological activities of the matrix, like signaling for cell adhesion and induction of migration, proliferation, and differentiation to achieve organ reconstitution and its functional remodeling [77,83].

Collagen, sulfated glycosaminoglycan, growth factors like BMP4, PDGF-BB, TGF-α, VEGF, TGFβ1, KGF, IGF-1, HGF, KGF, bFGF, or EGF can be preserved in the decellularization process [84]. For an appropriate preservation of the endogenous bioactive properties of the bladder acellular matrix (BAM), critical steps have to be included in the decellularization process, like, for instance, keeping the pH in the 7–8 range, avoiding heating, contamination, and freezing or lyophilization for long-term storage [79,85].

After decellularization, the complex reintroduction of cells must be achieved. This process is usually conducted within a bioreactor, which is able to simulate the in vivo environment for optimal cell growth, nutrition, and metabolism. However, cell type, cell number, and cell media play very important roles in ensuring a suitable environment for cell survival and differentiation [44]. New seeding techniques are under research to enable the replication of anatomically correct and physiologically functional bioengineered bladders [60,86]. Much progress has been made so far in stem cell engineering and urothelial differentiation. However, future studies will probably be focused on effective seeding methods for the promotion of angiogenesis and neural regeneration, which are both essential to successfully fabricate the ideal urinary reservoir [87].

#### 2.1.2. Natural Porous Polymer Scaffolds

Decellularized tissue scaffolds are commonly derived from porcine organs [74]. Pig and human bladders have anatomical and biological similarities, which render porcine-derived bladder cells an adequate model for tissue engineering strategies [88]. Native decellularized matrices employed for bladder substitution are typically derived from BAM [89] or from the small intestine submucosa (SIS) [90]. These scaffolds, consisting of fibrillar collagen type I and a porous basement membrane for recellularization, have been used either as grafts alone or seeded with urothelial cells (UCs) and smooth muscle cells (SMCs) [37,90,91] in diverse animal models [90,92,93] with some success. However, similar acellular tissue scaffolds failed in larger models such as pigs and dogs [91,94].

**Figure 3:**
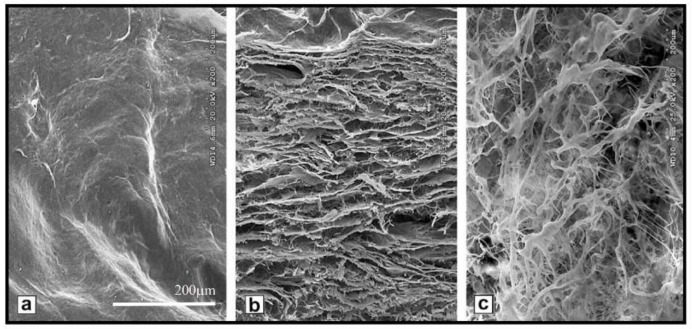
Acellular bladder submucosa scaffolds. Scanning electron micrographs (SEM) of fresh bladder submucosa (BSM) at 1000× magnification: surface (**a**) and cross section (**b**,**c**). The scale bar indicates 200 µm. Reprinted with permission from Elsevier Ltd., Liu et al. [58].

Very promising BAM porous scaffolds for urinary bladder regeneration are derived from the bladder submucosa (BSM) (see Figure 3) and subsequently recellularized with human bladder cells. The porosity of these scaffolds can be increased by performing a peracetic acid (PAA) treatment before decellularization to aid cell matrix penetration and promote cell growth in vivo [58].

Natural porous polymer scaffolds can be produced nowadays by many different methods, such as solvent casting, phase separation, gas-foaming techniques, electrospinning, freeze extraction, lyophilization, 3D printing, etc., to produce porous urinary bladder scaffolds with very different degrees of interconnected porosity and with a wide range of pore morphologies [34]. Thus, an interconnected porous natural type I collagen scaffold with pores ranging from 50 to 100 µm has been recently prepared by lyophilization and utilized as a strip clamped in a Bose Electroforce Bio-Dynamic bioreactor to study the effect of a cyclic uniaxial strain on urinary bladder cells [48].

Another important naturally occurring polymer is hyaluronic acid (HA), which has been utilized in many bioengineering applications, such as wound healing, growth factor release, and 3D bioprinting, among others [95]. Another renewable natural biomaterial, which is very promising for natural bladder scaffold after modification with cell-adherent peptides [96] and has also been approved by the U.S. Food and Drug Administration (FDA) for human wound healing [97], is alginate.

Tissue-engineered pericardium (TEP) is another promising natural scaffold biomaterial for bladder tissue engineering. Small-sized TEP samples have shown excellent results; however, further research must still be conducted with larger scaffolds [53].

#### 2.1.3. Collagen and Its Properties

The natural material derived from the ECM, collagen [98], exhibits minimal inflammatory and antigenic responses [99] and is a natural adhesive ligand that facilitates cell attachment and structural integrity, improving tissue regeneration. Thus, collagen has been approved for many types of medical applications, such as dermal wound and abdominal wall repair, by the FDA [100].

Even though collagen has been studied extensively in (pre)clinical bladder bioengineering because of its mentioned favorable properties, natural matrices based on it are hampered by the poor and slow SMC ingrowth after scaffold implantation [101].

### 2.2. Synthetic Bladder Scaffolds

Synthetic polymer scaffolds are also very attractive for bioengineering approaches for bladder regeneration because they can be produced at industrial scale, and their physical and chemical properties, such as degradation rate, compression performance, wettability, etc., can be tailored by synthetic reactions. However, not all synthetic polymers are valid for bladder bioengineering purposes [35]. Some synthetic polyesters, such as polyglycolic acid (PGA), polylactic acid (PLA), and copoly(lactic/glycolic) acid (PLGA), are biodegradable, biocompatible, non-toxic, and widely used in many diverse biomedical applications [102]. These thermoplastics have been also approved by the FDA for diverse clinical applications, such as sutures [103], because their degradation products are non-toxic [102]. Nowadays, synthetic PGA, PLA, and PLGA porous scaffolds can be fabricated with the desired interconnected morphology by a wide range of methods and technologies [102,104,105]. Thus, bladder-derived cells have been successfully cultivated onto PGA biodegradable scaffolds in the form of PGA meshes, molded into the shape of a bladder and PLGA surface-coated [37]. Nanofibrous poly-l-lactide (PLLA) scaffolds have been shown to be potentially useful for bioengineering approaches for bladder cancer patients requiring cystoplasty [56]. The mechanical properties of biodegradable poly(ε-caprolactone) (PCL) foam scaffolds and cell behavior on them showed that these scaffolds may be very suitable for bladder tissue engineering [59]. However, these biodegradable polymers lack bioactive factors that are present in acellular matrices, and thus their chemistry must be modified.

Other biomaterials that could be very favorable for bladder scaffold manufacture are polyanhydrides, poly (ortho-esters) [106], and silk fibroin [52]. In addition, compared to PGA and SIS, silk fibroin exhibited significant advantages for bladder augmentation in a mouse model [52], and silk scaffolds were shown to support primary and pluripotent cell responses pertinent to bladder tissue engineering [107].

### 2.3. Composite Bladder Scaffolds

**Figure 4:**
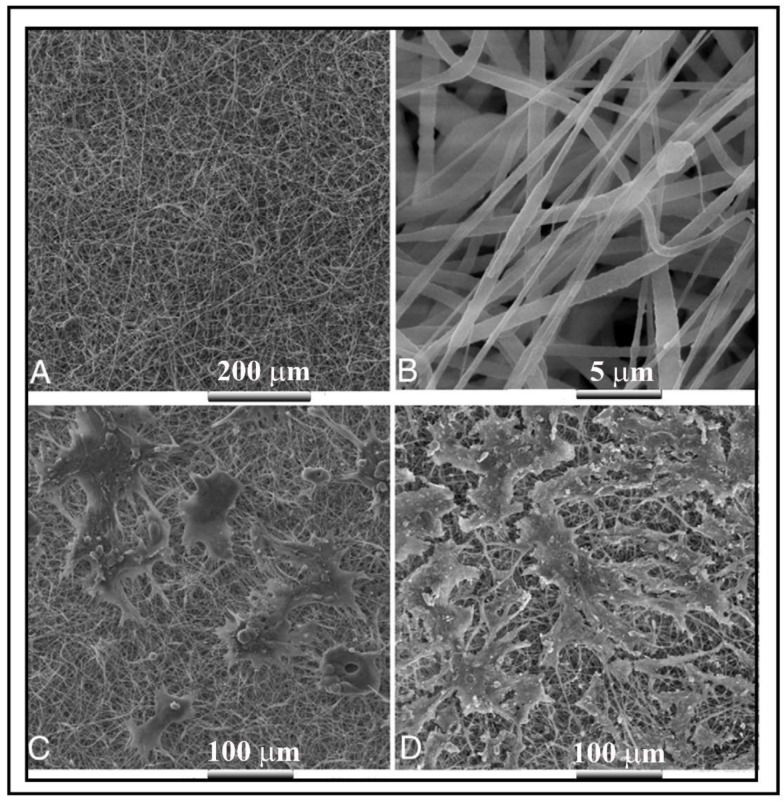
Morphology of a poly(ε-caprolactone (PCL)/poly-l-lactide (PLLA) scaffold and cell distribution by SEM. Non-woven and randomly oriented fibers of PCL and PLLA at 500× (**A**) and 5000× (**B**) magnifications; (**C**) Urothelial cells on the scaffold surface preserving their phenotype by creating their typical colonies; (**D**) Bladder smooth muscle cells expanded and proliferated on the scaffold. Reprinted with permission from Elsevier Ltd., Shakhssalim et al. [47].

Composite biomaterials can be very useful to tailor many physical and chemical properties required for bladder engineered scaffolds, which must be composed of diverse layers formed by different cell types and tissue structures. Composite scaffolds are fabricated with at least two different biomaterial systems and combine the properties of each component. Thus, for example, hybrid matrices composed of a PLGA mesh combined with collagen have been processed to obtain a sponge or a gel [108]. Biodegradable PGA scaffolds have also been improved in association with TEP [60] and collagen [109,110] acellular matrices. The optimization of cell attachment and in vivo bladder wall construction and increased compliance and capacities can be achieved through these associations. Other natural polymers, such as HA, can be added to the acellular matrix scaffold to render it impermeable [111]. Poly(ε-caprolactone)/poly(lactic acid) in the form of an electrospun scaffold (see Figure 4) recently promoted bladder regeneration in a canine model [47].

The potential use of plastic-compressed collagen-poly(lactic acid-co-ε-caprolactone) (PLAC) hybrids as engineered bladder scaffolds was reported and showed that human bladder smooth muscle and urothelial cells proliferated excellently in and on the composite, with a lower inflammatory reaction in vivo than PLAC scaffolds alone [51]. Electrospun-aligned PCL/PLLA nanofibrous scaffolds promoted cell differentiation in human bladder tissue engineering [112]. 3D synthetic composites have been also utilized to engineer urinary bladder smooth muscle cells from adipose stem cells [40]. Nanostructured polyurethane and PLGA composites have shown great promise to improve bladder tissue regeneration [49].

Although bioengineering approaches for bladder tissue are in their early development, all these studies demonstrate the rapid progress in the field during the last decades.

### 2.4. Stem Cells Approach

The stem cells approach is frequently employed in whole-organ regeneration because stem cells are undifferentiated cells able to replicate and differentiate into diverse kinds of cells. Stem cells have been extensively tested for cellular enrichment of the urinary tracts and vesical functional regeneration. Adult mesenchymal stem cells (MSC), can be isolated from different sources, including bone marrow and adipose tissue (adult-derived regenerative cells, ADRC), for autologous cell transplantation, already showing positive effects on the urethral functions in experimental animals [113,114] and safety and feasibility in humans [115]. MSC are capable to differentiate into several cell types, such as urothelium, fibroblasts, myoblasts, endothelial and smooth muscle cells [39,40], and even neurogenic cells [116]. ADRC are of special concern for promoting revascularization and neuronal and mesodermal regeneration. Thus, neural-differentiated ADRC possess glial characteristics and promote nerve regeneration, as observed in transplanted rat models [117]. Transplanted ADRC, in addition to providing a structural and functional support, secrete various angiogenesis-related cytokines, including vascular endothelial growth factor and hepatocyte growth factor [118] that induce, in fact, endogenous migration and regenerative activation. In the animal experimentation reports these cells exerted their effect in vivo mostly through secretion of paracrine factors, rather than by significant cell replacement, which in fact, supports a poor and short survival in vivo.

Additionally to MSC, another source of autologous cells that allows to avoid immune rejections is represented by adult urothelial cells and smooth muscle cells have been efficiently harvested from biopsy material, expanded extensively in culture, and reimplanted into the same host [37]. When autologous bladder cells cannot be harvested, pluripotent stem cells may be a promising alternative for bladder regeneration. In this respect, induced pluripotent stem cells (iPSCs) are an alternative source of pluripotent cells artificially generated by somatic cell reprogramming [119]. Besides, iPSCs can be generated from a wide range of somatic cell types of various species differentiated into various kinds of cells [120]. iPSCs can efficiently be directed into endoderm-specialized cells and can be induced to differentiate into bladder mature cells like urothelium cells, by expressing the master transcriptional regulators IFR1 and GATA4 [121], or to smooth muscle cells [122]. Alternatively, MSCs from iPSC were demonstrated to show less variability in cell maturation processes and in relation to the donor and tissue types, providing a better cell quality and undergoing more homogenous and more efficient differentiation process [107,123,124] (summarized in Table 3).

### 2.5. Role of Microenvironment Elements

Cells are strongly affected by their microenvironment elements: ECM, physical stimuli, factors, and nutrients. The main components of the ECM, which are continually changing, are structural proteins, like elastin or fibronectin, collagens, and space-filling proteoglycans arranged in a unique 3D structure [127]. The ECM allocates bioactive components, being a reservoir of growth factors and other nutrients directly linked to the structural protein network or embedded within the tissular fluid. The above-mentioned bioactive molecules orchestrate a cascade of functional signals, providing a 3D natural platform to regenerate the cellular structure [128]. The composition and architecture of the ECM produce a strong influence on the development, function, and protection against urine and pathogens [127]. Furthermore, the ECM strongly influences tissue regeneration [34]. UCs/SMCs co-cultures on ECMs can be achieved through a multilayered strategy to favor cell engraftment and differentiation [51,58].

### 2.6. Functionality

Although the formation of a bioengineered tissue with a morphology similar to that of the native bladder tissue has already been accomplished, the restoration of physiologic voiding using these constructs has not been achieved so far [42]. To solve this complex limitation, the development of correct muscle alignment, proper innervation, and vascularization is necessary, involving at the end a high specialization and maturation of all cell types and their interactions. Innervation is greatly affected by different neurotrophic factors and cellular and topographic features. Rapid neo-vascularization is critical in tissue-bioengineered constructs to ensure an adequate oxygen and nutrients supply for cell survival, proliferation [129], and adequate removal of harmful metabolites [130,131] and to achieve a complete restoration of organ structure and functionality. Useful methods that support early vascularization in bioengineered tissue include the application of angiogenic agents, such as VEGFs [132] and bFGF, and the implantation of vascular endothelial cells combined with SMCs and UCs in an engineered construct. Another important multifunctional factor is bFGF, which produced a significant angiogenesis improvement and inhibited graft shrinkage in a rat bladder augmentation model [133], promoting mitogenic activity of fibroblasts and endothelial cells and displaying neurotrophic and angiogenic properties.

The vascularization of bioengineered organs can be achieved by ex vivo approaches, such as cell sheet engineering, spheroid coculture, scaffold functionalization, modular assembly, and bioprinting or addressed in vivo by arteriovenous loops, polysurgery, and genetic manipulations [130]. Scaffold survival is profoundly dependent on the formation of a new vascular bed, and this process becomes more difficult with the increase of the construct size [48]. We believe that the lack of functionality of the engineered bladder tissue is a good reason to discourage researchers and explains somehow the low high-impact scientific production in this scientific area during the last decade (see Table 4). However, we firmly believe that bladder tissue bioengineered scaffolds with perfectly controlled morphology and capability to deliver multiple neural and angiogenetic factors will become a real option for physiologically functioning bladder constructs in the near future, although preclinical research on large animal models with defective bladders is necessary to optimize the methods of bioengineering bladder reconstruction in humans [134].

Congenital disorders, trauma, cancer, inflammation, infection, iatrogenic injuries, or any other condition of the genitourinary system can produce bladder damage, requiring often eventual reconstructive procedures. To try to solve this imperative need, the translation of extracellular matrix-based scaffolds into the clinic provides chances and challenges. Nevertheless, a thorough understanding of the cellular and molecular mechanisms underlying tissue regeneration is necessary, together with an increased knowledge of the extracellular matrix itself and as a potential carrier for the improvement of endogenous regeneration. In this respect, the interactions within the ECM, between the ECM and the ectopic integrated cells, and between these cells and the endogenous hosted tissue remain elusive, allowing us to explore a wide variety of alternatives. Bladder recellularization and urodynamic functional recovery have been successfully tested in small animals [135]. However, the translation into humans requires more efficient procedures ex vivo and a better understanding of the role played by the ectopic transplanted cells.

### 2.7. Animal Models for Translational Research

Despite the functional and structural regeneration of the bioengineered neobladders described in several preclinical studies in rodents [87,136], limited and so far non efficient clinical translation has been reported. First, porous matrices composed of a diverse range of biomaterials alone or forming a composite of natural and synthetic nature were used for urinary bladder regeneration, with a wide range of outcomes in several clinical trials, and failed to meet their expectations in most cases (reviewed in [134]). Additional investigation on translational models, increasing our knowledge of the mechanisms of cellular and functional regeneration in in vitro engineered bladders, should be expanded before further clinical applications on large animal models with defective bladders.

Recently, Gevaert et al. showed in detail the highly similar organization of the interstitial network in the upper and deep lamina propria and the detrusor muscle in human, rat, and mouse bladders [137]. Despite these extensive similarities of the interstitial phenotypes, several obvious interspecies differences exist, like a predominantly myoid phenotype and contractile micro-filament-enriched lamina propria in the human bladder in comparison with the predominantly fibroblast-like phenotypes found in rat and mouse bladders, which would affect in turn the functional regeneration process. Illustrative of this hypothesis is the results of the recently reported data of a clinical phase II trial for bladder augmentation, using a biodegradable scaffold previously seeded with autologous cells. All patients showed improved compliance, although not earlier than one year after transplantation and with no clinical or statistical improvements in bladder capacity and cellular reconstitution during the three years of the study. Indeed, adverse events occurred in all patients [31]. Additional experimentation has been conducted in the last decades for a better translation of the regenerative approaches in large animals. The side effects and complications of the xerograph tissue implantation were analyzed in a canine model in which porcine SIS was seeded with autologous smooth muscle cells, leading to an improved integration and quality of the transplanted tissue in the presence of the autologous transplanted cells [138].

## 3. Urinary Bladder Bioreactors

Bioreactors are sophisticated engineering simulation biosystems which can control environmental factors such as pH, temperature, and oxygen concentration and can be utilized to improve tissue regeneration by subjecting the blood vessels [139], cartilage [140] and ligaments [141] growing in a tissue to a variety of forces and stimuli: stress–strain, compression, pulsatile flow, shear stresses, and electrical stimulation. In the field of bladder tissue engineering, the simulation of the normal physiological functions of filling and emptying with an in vitro bioreactor may provide the additional mechanical performance required to enhance the functional outcome after implantation [41,61,142,143] and may accelerate tissue organization and maturation. In vivo bioreactors, which are the in vivo and ex situ system where the bioengineered construct is placed prior to implantation into the target location, constitute another promising bioengineering approach in the field of bladder regeneration. This in vivo preconditioning may further support tissue development, improve vascularization of the bioengineered tissue, and prevent fibrosis and contractility loss [42].

Thus, a very extensive search of the literature was conducted in order to find the most relevant publications and patents in the field of in vitro and in vivo bladder bioreactors during the last decade (see Table 4). It is remarkable that the scientific contribution to this field has been very poor. Thus, not even a Q1 paper has been published from 2012, and a recent book chapter from Elsevier [144] mentions only a bladder bioreactor developed in 2011 [55].

### 3.1. In Vitro Bladder Bioreactors

In the last decade, enormous progress has been achieved in the bioreactor technology field in order to devise bioreactors able to grow tissue-engineered vascular constructs in a physiological environment similar to that of the body, while maintaining an aseptic environment [41]. These studies have demonstrated that mechanical stresses can create a beneficial effect and can be even essential for growing tissue-engineered vascular constructs. However, many problems need still to be addressed. Thus, some studies on shear stress have shown that a high shear stress inhibits endothelial and smooth muscle cell proliferation [148]. In addition, the bioengineered tissues present different stages of development in vivo, and each stage requires different mechanical conditioning regimes, owing to the increased accumulation of ECM and to the developing structural organization [149].

The advantages of the in vitro bioreactor systems are their high experimental reliability and reproducibility, which are based on the excellent control and regulation of the cell culture parameters [150]. Besides, the bioreactors provide a versatile environment to study cell–scaffold interactions under different pressure conditions, which cannot be achieved by utilizing static cultures in plates or flasks [97,151].

One of the most important objectives of this research field is the improvement of the process of bladder tissue building, obtaining an impermeable epithelial surface to obtain well-differentiated UCs and SMCs and a suitable mechanically resilient and compliant ECM. Therefore, the first step usually consists in developing a urinary bladder bioreactor with an efficient process monitoring that recapitulates the in vivo bladder function in a reliable and reproducible way. Bioreactor design criteria must include the possibility of mechanical and nutrient environments control to promote cell proliferation, differentiation, and favorable matrix deposition [41].

One of the first bladder bioreactors was patented in 2007 (Patents CA2613945-A1 and US2009233361-A1) and consisted of two polycarbonate chambers separated by two interlocking rings [61]. This bioengineering system was devised to simulate normal urinary bladder dynamics in a tissue-engineered construct located between the two chambers. The results of this study suggested that gene expression could be mechanically simulated in a short period of time (e.g., collagens, caldesmon, uroplakin II), and that by this bioreactor was suitable for simulating the normal physiological bladder cycling ex vivo. This study demonstrates that bioengineering approaches with bladder bioreactors might enhance cell viability and proliferation by preconditioning cell-seeded scaffolds in a simulated physiologic environment [61].

Normal urinary bladder dynamics simulation applying cyclical pressure differences over the developing tissue constructs have been also performed with a pulsatile hydrostatic pressure bioreactor, using a porcine urinary bladder membrane (UBM) scaffold containing L929 fibroblasts [57]. The results of this study demonstrated L929 growth, adhesion, viability, morphological changes, and mitochondrial activity on the UBM.

Another bladder bioreactor developed in 2011 was constructed to replicate physiological bladder dynamics with a cyclical low-delivery pressure regulator in order to simulate the filling pressures of the human bladder [55]. In this work, significantly greater human UCs growth occurred on the porcine ECM scaffolds cultured in the bioreactor compared with conventional static laboratory conditions after three days. This bioreactor design permits also a cell-seeded scaffold to be positioned between two closed chambers filled with culture medium and be mechanically stimulated by manually adjusting the pressure conditions to replicate the cyclical bladder filling and emptying, within a period of hours and seconds, respectively.

**Figure 5:**
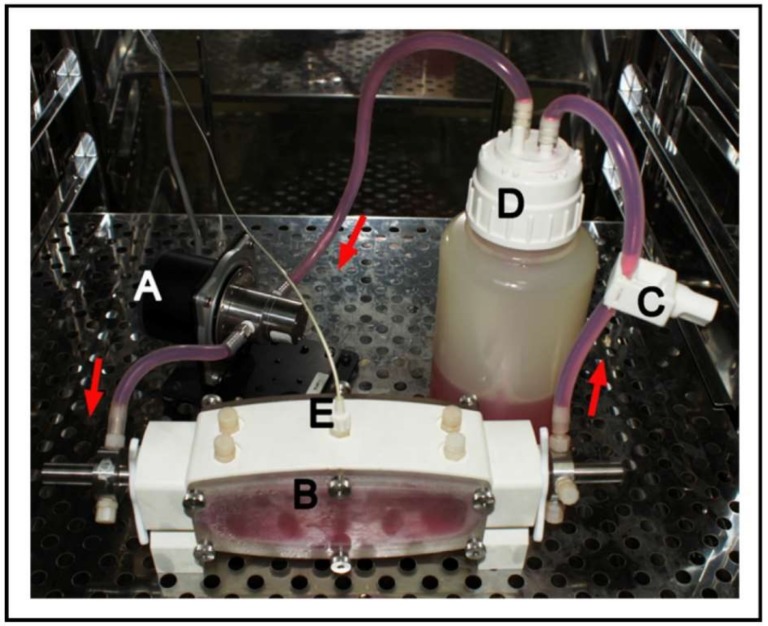
In vitro bioreactor: a computer senses the pressure in pressure chamber (**B**) by feedback via a pressure transducer (**E**). A computer interface establishes and maintains a specific hydrodynamic pressure by controlling a pump (**A**) output. A pressure valve (**C**) is almost completely closed to simulate bladder outlet obstruction. Dulbecco’s modified Eagle’s medium with 10% fetal bovine serum was used. The arrows indicate the flow direction. (**D**) Fluid reservoir. Reprinted with permission from Elsevier Ltd., Chen et al. [50].

Another study presented a bioreactor that mimicked the dynamic of bladder filling and emptying, delivering in this case a cyclic pressure increase up to 15 cm H_2_O, using fibroblasts and urothelial cells to obtain a reconstructed vesical equivalent (VE) [54]. The dynamic culture led to an urothelium profile like that of the native bladder, and the permeability analysis displayed a profile comparable to the native bladder. On the other hand, the first report of the use of cyclic hydrodynamic pressure stimulating the proliferation of human bladder SMCs cultured in scaffolds was published in 2012 [50]. Four pressures (0, 100, 200, and 300 cm H_2_O), controlled by a BOSE BioDynamic^®^ bioreactor, were applied to the cell-seeded constructs for 24 h (see Figure 5).

The purpose of a more recent study was to produce an optimum physiological stretch during the bladder cycle on a modified BOSE BioDynamic^®^ bioreactor, using human bladder smooth muscle cells (HBSMCs) seeded onto a silicone membrane, and demonstrated that the mechanical stretch can promote a magnitude-dependent morphological, proliferative, and contractile modulation of this type of cells in vitro [145].

Finally, one of the most recent contributions to the in vitro bladder bioreactor field studied the effect of a cyclic uniaxial strain on urinary bladder cells seeded on type I collagen scaffold strips [48], utilizing a BOSE Electroforce BioDynamic^®^ bioreactor. The dynamic stimulation of the SMC-seeded constructs resulted in cell alignment, enhanced proliferation rate, and differentiation of the SMCs to a more mature phenotype. However, the proliferation and differentiation of UCs were not improved by the mechanical stimuli. Therefore, despite the great advances achieved so far in this in vitro bioreactor research area for bladder tissue engineering, much more work has still to be conducted in order to find new strategies and designs to overcome all these problems.

### 3.2. In Vivo Bladder Bioreactors

**Figure 6:**
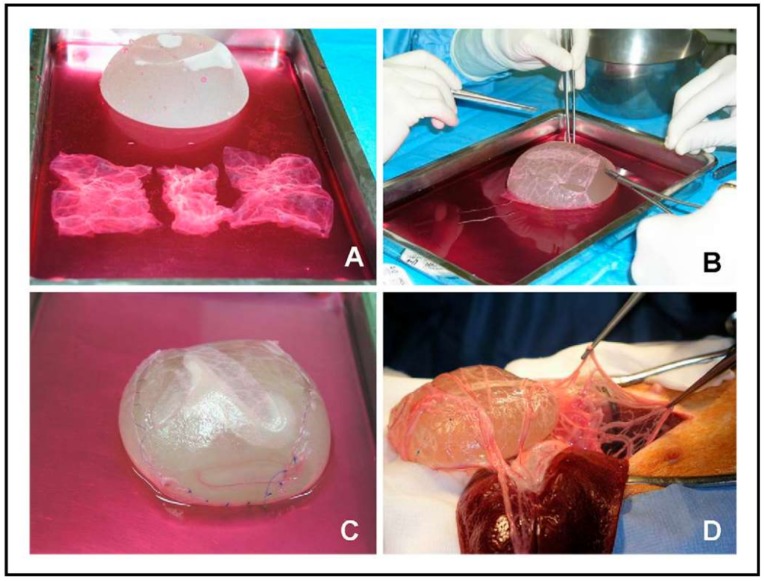
In vivo bioreactor: seeded scaffold preparation (**A**–**C**) and implantation (**D**) in the omentum. Reprinted with permission from Elsevier Ltd., Baumert et al. [147].

In vitro bioreactors have demonstrated to produce better tissue engineering constructs than the conventional static culture. However, the microvasculature of mature tissues cannot be simulated [147] in these systems, and, thus, fibrosis can easily occur in any type of constructs, likely due to ischemic damage during the transfer from the bioreactor system to the surgical location [152]. The idea of using omentum, which has a rich vasculature, as an in vivo bioreactor is probably the most important achievement in the field of bladder bioreactors during the last decade. In this work, published in European Urology in 2007 (see Table 4), scaffolds of small intestinal submucosa seeded with UCs and SMCs were transferred into the omentum of a porcine model (Figure 6) after three weeks of cell culture and were harvested three weeks later, showing that the scaffolds exhibited a multilayered urothelium and organized outer layers composed of SMCs and fibroblasts, with areas of dense vascularization [147]. Therefore, in vivo bioreactors have the advantage of inducing a rich vascularization, so that the scaffold can be transferred to the bladder replacement site without compromising the blood supply.

Another approach with many benefits over the conventional and current bladder tissue engineering strategies consisted in using the peritoneal cavity as an in vivo bioreactor [146]. In this work, bladder grafts showed 100% patency after 14 months and had developed a structure similar to that of the normal bladder. On the other hand, the potential of TEP, which is a collagen-rich matrix that promotes in vivo and in vitro tissue regeneration, as a source for the in vivo formation of bladder muscular wall grafts has also been evaluated [53]. This study, in which the bladder wall was used as an in situ bioreactor to mimic the natural environment supporting cell growth and differentiation, showed that utilization of the autologous bladder SMC seeding technique can improve the regeneration of the bladder wall. A more recent study investigated the potential of different scaffolds, using single- or multiple-layer biofilm scaffolds, TEP and PGA scaffolds, for in vivo bladder muscular and urothelial wall construction [60]. These different kinds of scaffold were placed in an in vivo bioreactor composed of two parts: the bladder mucosa and the seromuscular layer. The results of this study performed in this natural bioreactor showed that the PGA-coated TEP scaffold is a very promising porous biomaterial for muscular and urothelial fragment-seeding for bladder wall regeneration.

Scaffolds for tissue engineering are usually confined to small flat patches. However, large hollow urinary bladder organs made of a collagen-based scaffold with sites for ureters and urethra have been recently developed by a simple casting method [153]. Using the body as a bioreactor, in this study, porcine and also human bladder UCs and SMCs were capable to attach to the porous collagen-based matrix and maintain their phenotype.

Therefore, in vivo bioreactors demonstrate to be a long-sought alternative to traditional transplantation and provide a better replacement, congruent with the host immune system. However, this bioengineering strategy entails a two-stage procedure, and its advantages over the current tissue engineering approaches need to be further investigated before they can be translated into clinical practice.

## 4. Urinary Bladder Bioprinting

**Figure 7:**
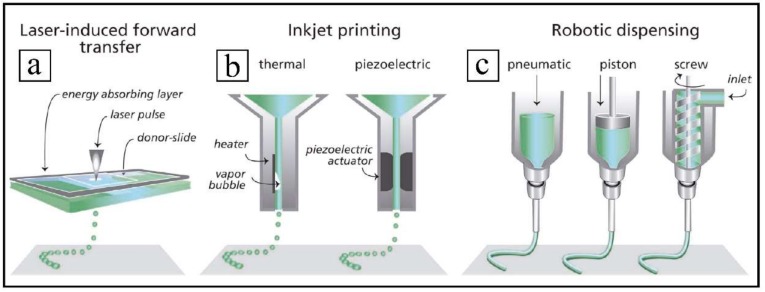
Bioprinting technologies: components of laser-induced forward transfer (**a**), inkjet printing (**b**) and robotic dispensing (**c**). Adapted from John Wiley and Sons, Malda et al. [163].

3D bioprinting is currently under intensive research to replicate functional transplantable organs with the great advantage of avoiding the deleterious host immune response. Besides, this sophisticated technology ideally renders possible to produce personalized organs in a perfectly controlled form. Clinical successes in this bioengineering field have only been achieved so far in more functionally simple organs, such as trachea, bronchi [67,154], blood vessels [155,156,157], and bladder [35,158,159,160]. Technical challenges related to the sensitivities of the living cells and tissue construction have to be addressed by integrating the multidisciplinary knowledge of a work team composed of experts in bioengineering, material science, biology, chemistry, physics, and medicine [161,162]. The main technologies employed for the deposition and patterning of biological materials are laser-induced forward transfer, inkjet printing, and robotic dispensing [163] (see Figure 7).

In 3D bioprinting research, the living cells are subjected to stress and biomechanical forces, which should be seriously considered because of their effects on cell vitality and differentiation [164]. It is very important to preserve cell vitality during the 3D bioprinting production process. Therefore, parameters such as production shape, speed, durability, or resolution of the bioprinted organ should be perfectly adjusted [165,166]. Despite these complex problems, much progress, regarding the utilization of natural hydrogels, such as collagen, alginate, and gelatin, as inks [167,168] and the integration of the sophisticated optical coherence tomography imaging technique [169] in the bioprinters, has been achieved so far. Therefore, we are confident that, step by step, bioprinting will be finally able to provide a real solution to the shortage of donors of bladders and other organs for transplantation in the near future.

## Figures and Tables

**Table 1:** Cost comparison by procedure received and type of urinary diversion. The cost presented include the total adjusted operating room and inpatient-related costs [30].

Type of Diversion	Robotic Radical Cystectomy Cost (US Dollars)	Open Radical Cystectomy Cost (US Dollars)
Neobladder	19,231.26	15,311.00
Ileal Conduit	18,388.19	16,648.58

**Table 2:** Types of urinary bladder scaffolds proposed in the last decade. The year, scaffold material, type of bladder scaffold, fabrication method, and references are indicated.

Year	Scaffold Material	Type	Fabrication Method	Reference
2017	Poly(ε-caprolactone)/poly(lactic acid)	Synthetic	Co-electrospinning	[47]
2017	Type I collagen	Natural	Lyophilization	[48]
2013	Polyurethane-poly-lactic-co-glycolic acid	Synthetic	Nanotechnology	[49]
2012	Polyvinyl alcohol (PVA)-based	Synthetic	Not specified	[50]
2011	Collagen-poly(lactic acid-co-ε-caprolactone)	Composite	Plastic compression	[51]
2011	Spun silk-based	Synthetic	Gel spinning	[52]
2011	Tissue-engineered pericardium (TEP)	Natural	None	[53]
2011	Autologous vesical equivalent (VE)	Natural	None	[54]
2011	Porcine extracellular matrix (ECM)	Natural	Decellularization	[55]
2010	Nanofibrous poly-l-lactide (PLLA)	Synthetic	Leaching	[56]
2010	Porcine bladder acellular matrix (BAM)	Natural	Decellularization	[57]
2009	Porcine bladder submucosa (BSM)	Natural	Decellularization/Oxidation	[58]
2009	Poly(ε-caprolactone) (PCL) foam	Synthetic	Water emulsion	[59]
2008	polyglicolic acid PGA-coated TEP	Composite	None	[60]
2008	Small intestine submucosa (SIS)	Natural	Commercially available	[61]

**Table 3:** Cell types employed for natural or synthetic bladder recellularization. The cell types seeded, tested models, main findings, and references are indicated.

Cells Seeded Type	Tested Model	Main Findings	Reference
Human bone marrow mesenchymal stem cells (BM-MSC)	Bladder augmentation with small intestine submucosa (SIS) in rats	1. Favorable short-term outcomes 2. Organized urothelium with increased basal cell layer staining and discrete muscle fascicles formation	[39]
Human adult derived regenerative cells (ADRC) differentiated into smooth muscle cells (SMC)	Bladder augmentation with co-poly(lactic/glycolic) acid (PLGA) dome composites in nude rats	1. Maintenance of bladder capacity and compliance for 12 weeks	[40]
Human bladder Urothelial cells (UC) and SMC	In vitro co-culture study in SIS	1. The layered coculture techniques resulted in organized cell sorting, formation of a well-defined pseudostratified urothelium, and multilayered smooth muscle cells with enhanced matrix penetration	[86]
Human BM-MSC	Bladder augmentation after partial cystectomy with bladder acellular matrix (BAM)	1. Recovery of nearly 100% normal bladder capacity for up to six months 2. Histological analyses demonstrated increased muscle regeneration	[125]
Rat SMC	Bladder augmentation by implantation of composite scaffolds of PLGA–BAM	1. The PLGA–BAM scaffold induced cell migration and proliferation and early revascularization	[126]
Porcine SMC and UC	Collagen I scaffolds in vitro culture study	1. Mechanical stimuli did not enhance or affect the proliferation and differentiation of urothelial cells; however, its preconditioning stimulus improved the proliferation and maturation of smooth muscle cells	[48]
Human induced pluripotent stem cells (iPSC) differentiated into UC and SMC	Silk-based scaffold coated with collagen types I or IV or fibronectin	1. Fibronectin-coated silk scaffolds facilitated iPSC differentiation toward both urothelial and smooth muscle lineages in vitro	[107]

**Table 4:** Publications and patents related to in vivo and in vitro bladder bioreactors in the last decade. The year, type of bioreactor, material support, cells used, journal name or patent code, last journal Impact Factor (IF), Quartile, and reference are indicated.

Year	Bioreactor	Support	Cells	Journal/Patent	IF 2016	Quartile	Reference
2017	In vitro	Type I collagen scaffold	Porcine UCs and SMCs	World Journal of Urology	2.743	Q2	[48]
2015	In vitro	Silicone membrane	Human bladder smooth muscle cells (HBSMCs)	World Journal of Urology	2.743	Q2	[145]
2012	In vitro	PVA-based scaffolds	HBSMCs	Journal of Urology	5.517	Q1	[50]
2012	In vivo	Pericardium biofilm and PGA scaffolds	UCs and SMCs	Tissue engineering Part A	3.485	Q2	[60]
2011	In vivo	Pericardium	Autologous SMCs	Journal of Pediatrics Urology	1.611	Q2/Q3	[53]
2011	In vitro	Autologous vesical equivalent	Fibroblasts and porcine UCs	Journal of Pediatrics Urology	1.611	Q2/Q3	[54]
2011	In vitro	Porcine ECM scaffolds	Human UCs	Urology	2.309	Q2	[55]
2010	In vitro	Porcine BAM	L929 fibroblasts	Artificial Organs	2.403	Q2/Q3	[57]
2008	In vitro	Porcine BAM and SIS scaffolds	UCs and SMCs	Tissue Engineering Par A	3.485	Q2	[61]
2008	In vivo	Myofibroblast-rich tissue capsules	Myofibroblast	J Tissue Eng and Regen Med	3.989	Q1/Q2	[146]
2007	In vitro	Porcine BAM and SIS scaffolds	UCs and SMCs	CA2613945-A1 US2009233361-A1	-	-	-
2007	In vivo	Porcine sphere-shaped SIS patches	Autologous UCs and SMCs	European Urology	16.265	Q1	[147]

## Abbreviations

3D	Tridimensional
ADRC	Adult-derived regenerative cells
BAM	Bladder acellular matrix
bFGF	Basic fibroblast growth factor
BM-MSC	Bone marrow mesenchymal stem cells
BMP4	Bone phosphogenic protein 4
BSM	Bladder submucosa
ECM	Extracellular matrix
EDTA	Ethylenediamine tetra-acetic acid
EGF	Epithermal growth factor
FDA	Food and Drug Administration
GATA4	GATA4 binding protein
HA	Hyaluronic acid
HBSMCs	Human bladder smooth muscle cells
HGF	Hepatic growth factor
IFR1	Interferon regulatory factor 1
IGF-1	Insulin growth factor 1
iPSCs	Induced pluripotent stem cells
KGF	Keratin growth factor
M	Million
MSC	Mesenchymal stem cells
PCL	Poly(ε-caprolactone)
PDGF	Platelet derived growth factor
PGA	Polyglicolic acid
PLA	Polylactic acid
PLAC	Collagen-Poly(lactic acid-co-ε-caprolactone)
PLGA	Co-poly(lactic/glycolic) acid
PLLA	Poly-l-Lactic
PS	Performance status
Q1	First quartile
SEM	Scanning electron microscopy
SIS	Small intestine submucosa
SMCs	Smooth muscle cells
TEP	Tissue-engineered pericardium
TGF	Transforming growth factor alpha
UBM	Urinary bladder membrane
UCs	Urothelial cells
VE	Vesical equivalent
VEGF	Vascular endothelial growth factor
